# Peroral Endoscopic Myotomy for Pediatric Achalasia: A Retrospective Analysis of 21 Cases With a Minimum Follow-Up of 5 Years

**DOI:** 10.3389/fped.2022.845103

**Published:** 2022-04-04

**Authors:** Dongzi Peng, Yuyong Tan, Chenjie Li, Liang Lv, Hongyi Zhu, Chengbai Liang, Rong Li, Deliang Liu

**Affiliations:** ^1^Department of Gastroenterology, The Second Xiangya Hospital of Central South University, Changsha, China; ^2^Research Center of Digestive Disease, Central South University, Changsha, China

**Keywords:** achalasia, pediatric, peroral endoscopic myotomy, POEM, long-term outcomes, endoscopic surgery

## Abstract

**Background:**

Peroral endoscopic myotomy (POEM) has shown promising short-term safety and efficacy in pediatric patients, while long-term outcomes are largely unknown. This study aimed to assess the clinical effects of POEM for pediatric achalasia who had a follow-up of at least 5 years.

**Methods:**

Pediatric patients from a single center who underwent a POEM between October 2011 and November 2016 were, respectively, collected and analyzed for long-term clinical outcomes. Patients were contacted to evaluate their current symptoms and encouraged repeat endoscopy and manometry. The clinical success, procedure-related parameters, adverse events, gastroesophageal reflux disease after POEM, and quality of life were evaluated.

**Results:**

A total of twenty-four patients who underwent POEM in our center were studied, with a mean age of 14.42 ± 2.65. Two of the 24 patients (8.3%) had previous treatment. The mean of the procedure time was 58.67 ± 19.10 min, 8.3% (2/24) of patients experienced perioperative adverse events. The current symptom scores were obtained from 21 patients at a mean follow-up of 92.57 months, the remainder were lost to follow-up after a mean of 38 months. Eckardt scores were significantly improved from preoperative baseline (preoperative 7.67 ± 1.62 vs. current 0.86 ± 1.28, *P* < 0.001). Long-term overall success was achieved in 95.8% of patients and none required retreatment for symptoms. 12.5% of patients were suffered from clinical reflux. 76.2% of patients expressed satisfaction with POEM. No severe adverse events were observed during the operation and the 5-years follow-up.

**Conclusion:**

POEM resulted in successful symptomatic mitigation in a majority of pediatric patients after 5 years. A multi-center large-scale, prospective study is necessary for a confirmed conclusion.

## Introduction

Achalasia is a rare esophageal motility disorder in pediatric, characterized by esophageal dysmotility and defective relaxation of the lower esophageal sphincter (LES). Its incidence in children is estimated to be 0.02–0.31 per 100,000 children per year (1–3). The pronounced symptoms of pediatric achalasia are dysphagia, regurgitation, vomiting, chest pain, weight loss, and respiratory symptoms (nocturnal cough, aspiration). If not appropriately treated, achalasia in children may lead to recurrent pneumonia, malnutrition, even intellectual and developmental disability. The symptoms may proceed into adulthood and impact quality of life.

Traditionally, achalasia in children has been treated with medical therapy, botulinum toxin injections, or balloon dilation, which do not have a long-lasting efficacy and often require additional interventions (4–8). Heller myotomy was recommended as first-line therapy for childhood achalasia, superior to balloon dilatation or botulinum injection (9, 10). After Inoue et al. described the first series of patients who received peroral endoscopic myotomy (POEM) in 2010 (11), POEM became a minimally invasive, rapid recovery, and preferred relatively novel procedure for achalasia in adults (12, 13). Multiple studies have also confirmed its efficacy and safety in children and adolescent patients (14–21). However, the long-term follow-up study in children, notably more than 5 years, is unclear.

In the present study, we investigated the clinical data of patients with pediatric achalasia who were at least 5 years out from their POEM procedure in our institution. We attempted to assess the endurance of symptom relief after POEM, as well as the outcomes of repeated esophagogastroduodenoscopy (EGD) and manometry, to understand the long-term outcome of the operation better.

## Materials and Methods

### Patients

The study was approved by the ethics committee of the Second Xiangya Hospital of Central South University. All the pediatric patients with achalasia who underwent POEM in our department between October 2011 and November 2016 were retrospectively collected. The inclusion criteria in this study included: (1) Achalasia diagnosed by Eckardt score ≥ 4 and further confirmed by esophagogastroduodenoscopy (EGD), barium esophagram, and/or esophageal manometry; and excluding others secondary to tumor, autoimmune diseases, etc. (2) patients with age ≤ 17 when performing POEM; (3) patients received POEM as a treatment more than 5 years. Those patients with severe cardiopulmonary disease, blood coagulation disorders, or other underlying diseases were excluded from this study. Demographics, presentation, clinical history, operation records, postoperative adverse events were retrieved from the medical records. Written informed consent was obtained from all patients’ parents or caregivers before the procedure was performed. All of them were informed of possible adverse events and other possible treatment options.

### Peroral Endoscopic Myotomy Procedure

Pediatric POEMs were performed by three experienced endoscopists with more than 30 adult POEMs (DLL, LL, HYZ). The patients were required to fast for 12 h before their procedures. POEM was performed under general anesthesia *via* tracheal intubation using a standard single-channel endoscopy (GIF-Q260J; Olympus, Tokyo, Japan) with a transparent cap (D-201-11,802, Olympus) attached to the front. A carbon dioxide insufflator (UCR, Olympus) was used as the air supply from the endoscopy. Other equipment and accessories included a high-frequency generator (ICC 200; Erbe, Tübingen, Germany), argon plasma coagulation unit (APC300; Erbe), injection needle (NM-4L-1; Olympus), hybrid knife (Erbe), dual knife (KD-650L; Olympus), and hemostatic clips (HX-600-135; Olympus).

**FIGURE 1:**
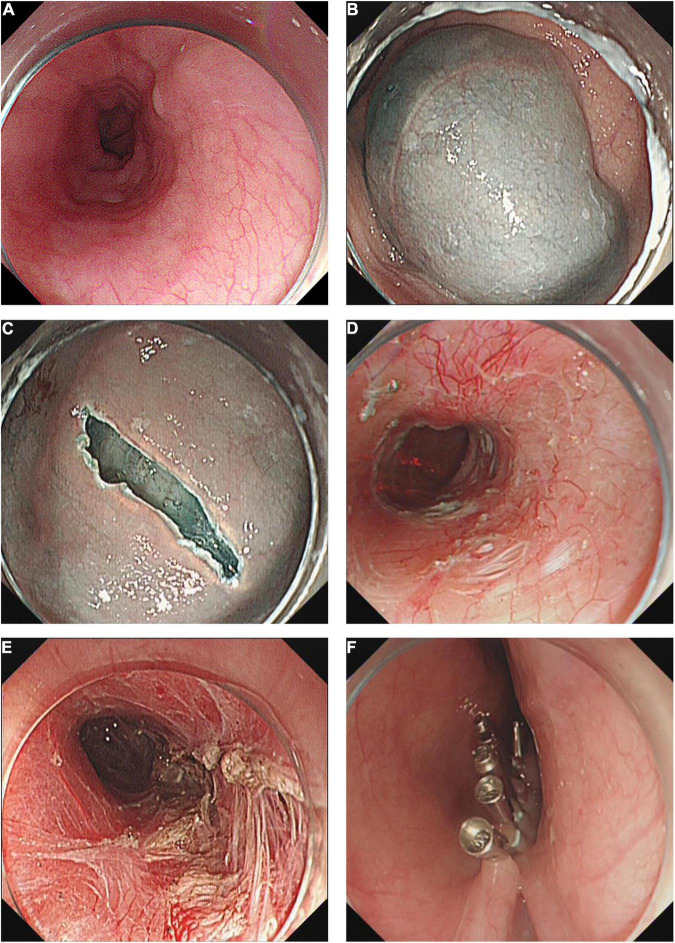
Technique of peroral endoscopic myotomy. **(A)** A dilated esophagus. **(B)** Submucosal injection. **(C)** Create tunnel entry. **(D)** Submucosal tunnel. **(E)** Circular myotomy. **(F)** Close the tunnel entry with metal clips.

The POEM procedure was performed as previously reported (16, 17): Briefly, this procedure included five major steps: (1) A submucosal injection was made using mixed solution (100 ml saline + 2 ml indigo carmine + 1 mg epinephrine), (2) A longitudinal mucosal incision (about 2–3 cm long) was made using a dual or hybrid knife; (3) A submucosal tunneling was created passing over the esophagogastric junction (EGJ); (4) Myotomy was started from 2 to 3 cm below tunnel entry, (5) After careful hemostasis, closure of mucosal incision was finished with metal clips. The right posterior approach at 6–10 cm above EGJ was generally applied except for some complicated cases, such as fibrosis of the posterior location. For patients who have previously undergone endoscopic or surgical treatment, tunneling and myotomy were performed in areas of normal tissue to avoid fibrosis or adhesions caused by previous treatment. Figure 1 describes the procedure of POEM.

Patients were fasted for 24 h after POEM, a liquid diet for 3 days, and returning gradually to a regular diet within 2 weeks. Intravenous proton pump inhibitor (PPI) and antibiotics were continued for 3 days. At Day 2 post-procedure, thoracoabdominal X-ray, or sometimes a chest CT, was performed to check for the occurrence of emphysema, pneumothorax, pneumoperitoneum and pleural effusion, etc.

### The Primary Outcomes

The primary outcome was clinical success defined as a post-treatment Eckardt score reduced to ≤ 3 and freedom from re-intervention for persistent or recurrent symptoms (12, 22). The Eckardt score is a sum of the symptoms for dysphagia, regurgitation, chest pain, and weight loss. The maximum score is 12, suggesting the most pronounced symptoms.

### The Secondary Outcomes

The secondary outcomes included technical success, procedure-related parameters, rate of adverse events, length of postoperative hospital stay, gastroesophageal reflux disease after POEM, and quality of life.

Technical success was defined as the completion of the whole POEM procedure. Mild perioperative adverse events (AEs) were defined as insufflation-related AEs, mucosal injuries, bleeding, pain requiring analgesics, and aspiration pneumonia; severe AEs were defined as delayed mucosal barrier failure, esophageal leaks, POEM related cardiopulmonary disease, intensive care unit admission, and conversion to a laparoscopic or open procedure (23). Length of postoperative hospital stay was defined as the number of days from procedure to hospital discharge. A GerdQ score of gastroesophageal reflux disease with a score of > 7 was defined as symptomatic reflux (18, 24–26). Reflux esophagitis was diagnosed and graded by EGD based on Los Angeles (LA) Classification. Clinical reflux was diagnosed if both symptomatic reflux and reflux esophagitis were positive. Quality of life (QoL) was assessed by Urbach scale questionaries, which is a 10-item measure of disease-specific health-related QoL that sampled the concepts of food tolerance, dysphagia-related behavior modifications, pain, heartburn, distress, lifestyle limitation, and satisfaction (27).

### Follow Up

Children with achalasia received follow up postoperatively at 6, 12, 24, and 36 months, and then every 2 years. Eckardt score was regularly obtained to assess clinical symptomatic response or recurrence *via* telephone or face-to-face visit. At 6 months postoperatively, EGD, esophageal manometry, and barium esophagram were recommended for outcome evaluation.

Patients who underwent POEM for more than 5 years at the current study were contacted *via* telephone to obtain a current Eckardt score, GerdQ score, and Urbach scale questionaries. Those symptoms were recorded from the patient’s self-report or their parents and caretakers. Meanwhile, Patients were encouraged to have a repeat EGD, barium esophagram, and esophageal manometry at the current study time in our hospital. Patients or caretakers who lived far from our hospital or were unwilling to return for follow-up were followed through detailed telephone interviews to assess for AEs and obtain a current physical condition, including questionaries about their symptoms and examinations and treatments at other hospitals.

During the follow-up, orally PPIs were only used in those with symptoms of reflux and/or endoscopy-proven reflux esophagitis.

### Statistical Analysis

The data were analyzed using SPSS 24.0 (Chicago, United States). Quantitative variables were presented as mean ± SD or medians and range. They were compared using a *t*-test or Mann-Whitney *U*-test. Categorical variables were expressed as absolute or relative frequencies. Those data were calculated using Chi-square or Fisher’s tests. A two-sided *p* < 0.05 was considered statistically significant.

## Results

### Patient Characteristics and Perioperative Outcomes

A total of 24 patients were collected during the specified duration. Fourteen of them were male and ten were female, with a mean age of 14.42 ± 2.65 year-old. The mean preoperative height is 1.54 ± 0.18 m and the mean preoperative weight is 42.52 ± 10.73 Kg. The median duration of symptoms is 14.50 (range 3–84) months. Two patients (8.3%) had received prior treatment, including 1 Heller myotomy and 1 balloon dilation. Fifteen patients underwent preoperative manometry. According to the Chicago classification of Esophageal Motility Disorders, the subtypes were type I 4 (26.7%), type II 10 (66.7%), and type III 1 (6.7%). The mean LES pre-POEM pressure was 30.42 ± 8.27 mmHg. The mean pre-POEM esophageal diameter was 46.11 ± 9.11 mm. One child who had a history of balloon dilation performed an anterior approach. Twenty-three children performed a right posterior approach. The median length of the submucosal tunnel is 12 (range 5–13) cm. The median incision length of esophageal, gastric and total is 6 (range 3–8) cm, 3 (range 2–3) cm, and 9 (range 5–10) cm, respectively. Full-thickness myotomy was performed in 50% (12/24) of the children. The mean operative time was 58.67 ± 19.10 min. The mean length of postoperatively hospital stay was 6.42 ± 2.15 days and the technical success of POEM is 100%(24/24). Two (8.3%) children developed perioperative subcutaneous emphysema. One children’s CT showed retroperitoneal, abdominal, and esophageal gas accumulation; another showed chest subcutaneous emphysema. Their gas-related AEs were spontaneously absorbed without any intervention. No severe perioperative adverse events occurred in those patients. The mean of total follow-up time was 85.75 ± 25.91 months (Table 1).

**TABLE 1:** Characteristics of the children who underwent peroral endoscopic myotomy.

Variable	Number
Total number	24
Age, mean ± SD, year	14.42 ± 2.65
Gender, n(%)	
Male	14 (58.3%)
Female	10 (41.7%)
Preoperative height, mean ± SD, m	1.54 ± 0.18
Preoperative weight, mean ± SD, Kg	42.52 ± 10.73
Duration of symptoms, median (range), month	14.5 (3–84)
Prior interventions, n(%)	2 (8.3%)
Achalasia subtype, n(%)	
Type I	4/15 (26.7%)
Type II	10/15 (66.7%)
Type III	1/15 (6.7%)
Preoperative LES pressure, mean ± SD, mmHg	30.42 ± 8.27
Preoperative esophageal diameter, mean ± SD, mm	46.11 ± 9.11
Length of submucosal tunnel, median (range), cm	12 (5–13)
Length of myotomy, median (range), cm	
Esophageal	6 (3–8)
Gastric	3 (2–3)
Total	9 (5–10)
Full-thickness myotomy, n(%)	12/24 (50%)
Procedure time, mean ± SD, min	58.67 ± 19.10
Length of postop hospital stay, mean ± SD, day	6.42 ± 2.15
Technical success rate of POEM, n(%)	24 (100%)
Perioperative AEs, n(%)	2/24 (8.3%)
Total follow-up period, mean ± SD, month	85.75 ± 25.91

### Symptomatic Outcomes

Of a mean follow-up period of 85.75 ± 25.91 months, 3 patients were lost for long-term follow-up. None of them had a history of prior treatment for achalasia. They were similar in age (14.29 ± 2.81 vs. 15.33 ± 0.58, *p* = 0.534), sex (57.1 vs. 66.7% of male, *p* = 1.000), and Eckardt Score at baseline (7.67 ± 1.62 vs. 7.67 ± 3.22, *p* = 1.000) compared to those who remained under study. With a mean follow-up duration of 38 ± 7.55 months, the mean Eckardt score at the last follow-up was 0.67 ± 0.58. These three patients were considered to be a clinical success at the previous follow-up. All patients have a 95.8%(23/24) clinical success rate, none underwent operational intervention again. With a mean follow-up time of 92.57 ± 19.38 months, the Eckardt score after 5 years of follow-up was significantly declined compared with the score before POEM (7.67 ± 1.62 vs. 0.86 ± 1.28, *p* < 0.001). Table 2 shows the change in each symptom component of the Eckardt score. One of the patients (Eckardt score = 4) suffered from mild dysphagia and chest pain from 39 months post-POEM. He needed to drink water when swallowing hard food most of the time, but still within tolerable limits. The chest pain can be alleviated by oral PPIs. The recent upper endoscopy showed multiple esophageal polyps (POEM related). Currently, he is undergoing symptoms and physiologic surveillance. The mean weight and height gain in patients are 17.41 ± 9.75 Kg and 0.15 ± 0.12 m in 5 years follow-up, respectively. At 5-year follow-up, the BMI was significantly increased from pre-POEM (preoperative 17.72 ± 1.97 vs. current 21.00 ± 2.19, *p* < 0.001). Additionally, Urbach scale questionaries were obtained from 21 patients to evaluate their quality of life. The median score was 14 (range 11–22). The outstanding symptoms reported by patients were the need to drink water while eating (14/21, 66.7%), bothered by the eating time (17/21, 81.0%). 71.4%(15/21) patients reported no limitation of lifestyle because of achalasia, 9.5% (2/21) of patients are very satisfied and 66.7%(14/21) are satisfied with their health as for achalasia.

**TABLE 2:** Comparison of clinical response outcomes pre and postoperatively (*n* = 21).

	Preoperative	> 5 Years postoperative	*P*
Dysphagia	2.86 ± 0.36	0.38 ± 0.59	<0.001
Regurgitation	2.48 ± 0.68	0.24 ± 0.44	<0.001
Chest pain	0.86 ± 0.96	0.24 ± 0.54	0.002
Weight loss	1.48 ± 0.98	0.00 ± 0.00	<0.001
Eckardt score	7.67 ± 1.62	0.86 ± 1.28	<0.001
BMI, mean ± SD, Kg/m^2^	17.72 ± 1.97	21.00 ± 2.19	<0.001
Esophageal diameter, mean ± SD, mm	49.00 ± 4.78	23.78 ± 3.87	0.001
LES pressure, mean ± SD, mmHg	30.29 ± 8.61	9.54 ± 1.85	<0.001

### Objective Outcomes

A total of 4 patients had paired barium esophagram results, the esophageal diameter is significantly decreased at 5 years post-POEM compared with the pre-POEM (49.00 ± 4.78 mm vs. 23.78 ± 3.87 mm, *p* = 0.001). A total of 10 patients had paired manometry results, and the LES pressure is significantly decreased at 5 years post-POEM compared with the pre-POEM (30.29 ± 8.61 vs. 9.54 ± 1.85 mmHg, *p* < 0.001) (Table 2). None of the children developed severe adverse events during the follow-ups, such as delayed bleeding, gastrointestinal fistulas, and secondary mediastinal or abdominal infections.

### Comparison of Outcomes Between Short-Term and Long-Term Follow-up

**FIGURE 2:**
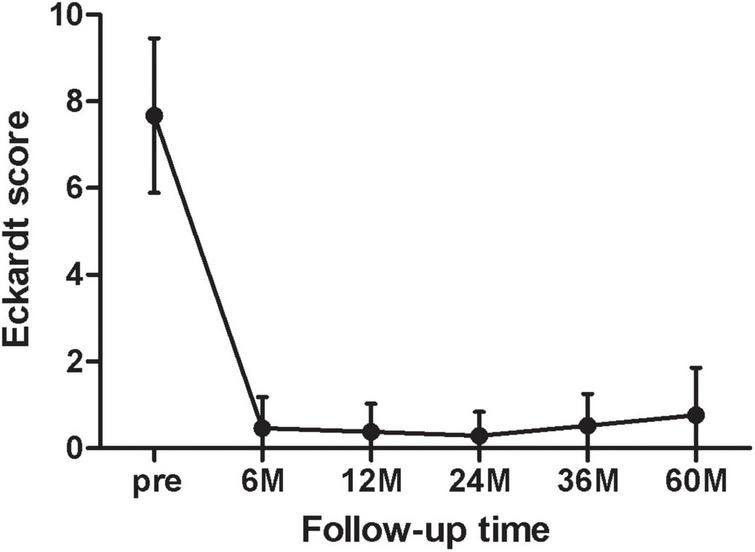
The mean of the Eckardt score at each follow-up point (6, 12, 24, 36, and 60 months).

Figure 2 showed the mean of the Eckardt score at each follow-up point (6, 12, 24, 36, 60 months). At 6 months postoperatively, the Eckardt score was significantly decreased from baseline (pre 7.67 ± 1.79 vs. 6 months0.46 ± 0.72, *p* < 0.001, *n* = 24). However, the Eckardt score began to gradually deteriorate in the second year (24 months 0.19 ± 0.40 vs. 60 months 0.76 ± 1.09, *p* = 0.019, *n* = 21).

With 3 patients having paired barium esophagram results and 9 patients having paired esophageal manometry results, there were no significant differences in Eckardt score, LES pressure, and esophageal diameter between 6 months and 5 years postoperatively (Supplementary Table 1).

### Post-peroral Endoscopic Myotomy Gastro-Esophageal Reflux

**FIGURE 3:**
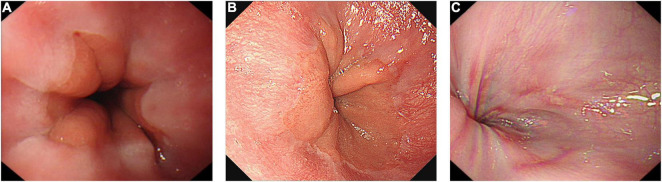
Postoperative endoscopy with a minimum follow-up of 5 years. **(A)** No abnormality. **(B)** Reflux esophagitis (Los Angeles Type A). **(C)** Reflux esophagitis (Los Angeles Type B).

At 6 months follow-up, 3 children (3/24, 12.5%) showed esophagitis on EGD (2 Los Angeles Type A, 1 Los Angeles Type B), their symptoms relieved after a 4-week oral PPI therapy. At 5 years follow-up, five patients (5/21, 23.8%) were suffered from symptomatic reflux, three patients (3/16, 18.8%) showed reflux esophagitis (2 Los Angeles Type A, 1 Los Angeles Type B) on endoscopy, two patients (2/16, 12.5%) were suffered from clinical reflux (Figure 3). No cases of Barrett’s esophagus were found. One patient with Gerd Q > 7 didn’t perform endoscopy at the end of our study. Eight patients received oral PPI therapy for 4 or 8 weeks during the follow-up.

## Discussion

In the present study, our result mainly indicated that POEM provided satisfactory long-term safety and efficacy for pediatric patients with achalasia. These data add to a growing body of evidence, much of which comes from the adult population. In the 10 years since POEM was performed, many studies have shown POEM resulted in a durable long-term symptomatic relief for 83–93% of adult patients with achalasia (28, 29). Previous studies have indicated that POEM has a short-term benefit in children patients, with a median follow-up duration of about 13.2–40 months (14–20). Pediatric patients assume a longer life expectancy, the long-term therapeutic effect for them is particularly crucial. However, owing to the low incidence of pediatric achalasia, the long-term efficacy of POEM in children is vague. In our study, we retrospectively reviewed 21 pediatric patients and performed a follow-up with a mean duration of 92.57 months, ranging from 60 to 122 months. To the best of our knowledge, this is the first study to indicate the safety and efficacy of POEM for pediatric achalasia with a minimum follow-up of 5 years.

In our research, all the pediatric patients with preoperative dysphagia and regurgitation reported improved or resolved symptoms at 5-years follow-up. All of them were free from the further intervention for achalasia after POEM. The total long-term clinical success rate was 95.8%. This good outcome of POEM in children suggested superior to that in Heller’s myotomy and balloon dilatation (30, 31). Children tend to have better treatment outcomes than adults, compared with a 5-year long-term success rate of 80–83% for adults (28, 29). This may be attributed to persistent eating disorders that can cause developmental delays in children, they earned more attention than adults, and their LES structure may be more malleable. The mean Eckardt score at each follow-up point showed a gradual and slight worsening of symptoms in pediatric patients beginning in the second year. A similar trend can be seen in the long-term follow-up study of adult POEMs (28). Success rates for balloon dilation and Heller myotomy also showed a gradual decline in a randomized controlled study (32). All of these results indicate the progression of achalasia symptoms requires careful monitoring and evaluation. Noteworthy, besides weight loss, failure to gain weight is an important aspect in assessing the severity of achalasia in pediatrics. Therefore, we recommend that physicians pay attention to growth indicators in children with achalasia.

It is well known that prior treatment may induce submucosal fibrosis, increasing the technical difficulty of subsequent myotomy and thus increasing the time of surgery and the risk of severe AEs. For the patient with treatment history, we performed an opposite myotomy avoiding the previous myotomy site or cutting the fibrotic tissue; details have been elaborated in our earlier research (33). In the present study, two patients who received prior treatment had significant symptoms of remission and reduced LES pressure after performing POEM and were satisfied with their life quality at present. Although one patient who underwent previously Heller myotomy had reflux (Gerd Q = 12) and esophagitis on gastroscopy at 5 years follow-up, the symptoms were relieved with an 8-week oral PPI and Eckardt score was 3. This result extends those from previous studies (34) and provides additional evidence in children that POEM has an excellent long-term efficacy for more than 5 years as a remedial treatment.

Moreover, the POEM procedure is relatively complicated, and it is perhaps more difficult in the pediatric population. In our experience, there are several key points to the success of pediatric POEM. Above all, the operator should be an expert with rich experience in endoscopic submucosal dissection, who can skillfully operate endoscopes and deal with possible complications. It would be better the operator had experience in adult POEM before performing pediatric POEM. Secondly, the operation should be performed as early as possible before significant esophageal dilation or distortion develops. Thirdly, submucosal injection into the esophageal cavity with a mixed solution containing indigo can be used to preset tunnel routes to ensure a straight tunnel into the proximal stomach. Fourthly, intraoperative mucosal integrity should be maintained and metal clips would be used if necessary.

Gastroesophageal reflux disease (GERD) is the most concerning complication after POEM in consideration of it does not include antireflux procedure as is typically performed during Heller’s myotomy. In our study, the 5-year long-term consequences of clinical reflux, symptomatic reflux, and esophagitis arising after POEM are 12.5, 23.8, and 18.8%, respectively, which was comparable to Chen’s result, who found the overall clinical reflux adverse event rates were 19.2% in children (14). In a recent study, Nabi et al. found erosive esophagitis was detected in 55% of children at 3 months post-procedure (19). This difference may be related to GERD subjective and objective measurements. Additionally, pediatric patients are received more attention from parents and doctors. They were placed on medical therapy if they had evidence of GERD; thus, the incidence of GERD may be controlled in long-term follow-up. Long-term surveillance endoscopy after treatment for achalasia showed that those patients may develop erosive esophagitis, Barrett’s esophagus, and even esophageal adenocarcinoma (28, 35, 36). These results indicated routine surveillance for GERD after POEM should be recommended in pediatric achalasia as well. In our study cohort, no dysplasia or malignancy was identified yet. However, follow-up over decades, even life-long, is warranted in future studies.

At the study time, most patients were over 16 years old. Thence, the Urbach Scale was applied to assess patients’ long-term quality of life *via* face-to-face visits or phone contacts. The only adolescent patient had completed the QoL questionnaire with the help of parents. Majority of the patients in whom at least 5 years had elapsed since POEM are satisfied with their health regarding achalasia. POEM could result in a good adult quality of life for pediatric patients.

Several factors could limit the extent to which the results can be generalized in our study. First of all, our sample size was small and the mean age was slightly older. This shortage is due to achalasia being less common in children than adults, and younger children tend to receive treatment in children’s hospitals in China. Besides, despite these operations being performed by our most experienced endoscopist, children included still present our initial experience with the procedure. According to the POEM learning curve (37), The results could change as the number of surgeries increases. Third, although we emphasized the importance of long-term monitoring for achalasia in children, some patients were not reviewed by endoscopy and manometry. None of them were willing to take a 24-h PH test. Therefore, the incidence of reflux esophagitis is likely to be misestimated.

In summary, peroral endoscopic myotomy could lead to a favorable long-term efficacy in most childhood achalasia and little need for renewed intervention. Future, a multi-center prospective study with a large sample size is required.

## Data Availability Statement

The original contributions presented in the study are included in the article/Supplementary Material, further inquiries can be directed to the corresponding author/s.

## Ethics Statement

The studies involving human participants were reviewed and approved by the Ethics Committee of the Second Xiangya Hospital of Central South University. Written informed consent to participate in this study was provided by the participants’ legal guardian/next of kin. Written informed consent was obtained from the individual(s), and minor(s)’ legal guardian/next of kin, for the publication of any potentially identifiable images or data included in this article.

## Author Contributions

DP designed the study protocol, and took part in the collection of data and writing the manuscript. YT shared in designing the study protocol, data collection, and manuscript revision. CJL shared in the collection of data and statistical analysis. LL, HZ, CBL shared in the description of detailed operation steps and picture collecting. RL and DL conceived the original idea, supervised the project, and contributed to all phases of the article. All authors contributed to the manuscript and approved the submitted version.

## Conflict of Interest

The authors declare that the research was conducted in the absence of any commercial or financial relationships that could be construed as a potential conflict of interest.

## Publisher’s Note

All claims expressed in this article are solely those of the authors and do not necessarily represent those of their affiliated organizations, or those of the publisher, the editors and the reviewers. Any product that may be evaluated in this article, or claim that may be made by its manufacturer, is not guaranteed or endorsed by the publisher.
