# Supplementary material for: Peroral Endoscopic Myotomy for Pediatric Achalasia: A Retrospective Analysis of 21 Cases With a Minimum Follow-Up of 5 Years
Source: Front Pediatr. 2022 Apr 4;10:845103. doi: 10.3389/fped.2022.845103 (PMC9013798; doi:10.3389/fped.2022.845103)
Supplement: Supplementary file 1 [file Table_1.DOCX]

**Supplemental Table 1 Comparison of clinical response outcomes 6 months and 5 years postoperatively**

|  | 6 months postoperative | >5 years postoperative | *P* |
| --- | --- | --- | --- |
| Dysphagia | 0.19±0.40 | 0.38±0.59 | 0.214 |
| Regurgitation | 0.14±0.36 | 0.24±0.44 | 0.162 |
| Chest pain | 0.05±0.22 | 0.24±0.54 | 0.104 |
| Weight loss | 0.00±0.00 | 0.00±0.00 | - |
| Eckardt score | 0.38±0.67 | 0.86±1.28 | 0.086 |
| esophageal diameter, mean±SD, mm | 29.00±2.86 | 25.63±1.31 | 0.068 |
| LES pressure, mean±SD, mmHg | 12.2±2.87 | 9.70±1.90 | 0.065 |
